# BAM15 Relieves Neurodegeneration in Aged *Caenorhabditis elegans* and Extends Lifespan

**DOI:** 10.3390/metabo12111129

**Published:** 2022-11-17

**Authors:** Injeong Cho, Hyun-Ok Song, Ha Eun Ji, Sungtae Yang, Jeong Hoon Cho

**Affiliations:** 1 Department of Biology Education, College of Education, Chosun University, Gwangju 61452, Republic of Korea; 2 Department of Infection Biology, Wonkwang University School of Medicine, Iksan 54538, Republic of Korea; 3 Department of Microbiology, School of Medicine, Chosun University, Gwangju 61452, Republic of Korea

**Keywords:** BAM15, mitochondrial uncoupler, neurodegeneration, aging, *C. elegans*

## Abstract

BAM15 was recently screened as a protonophore uncoupler specifically for the mitochondrial membrane but not the plasma membrane. It is equally as potent as FCCP, but less toxic. Previously, mitochondrial uncoupling via DNP alleviates neurodegeneration in the nematode *Caenorhabditis elegans* during aging. Therefore, we investigated whether BAM15 uncouplers could phenotypically and functionally reduce neuronal defects in aged nematodes. We observed green fluorescence protein-tagged mechanosensory neurons and performed touch and chemotaxis assays during aging. Wild-type animals treated with both 50 µM BAM15 and 10 µM DNP showed reduced mechanosensory neuronal defects during aging, which correlates with the maintenance of touch responses and short-term memory during aging. Uncoupler mutant *ucp-4* also responded the same way as the wild-type, reducing neurodegeneration in 50 µM BAM15 and 10 µM DNP-treated animals compared to the DMSO control. These results suggest that 50 µM BAM15 alleviates neurodegeneration phenotypically and functionally in *C. elegans* during aging, potentially through mitochondrial uncoupling. In accordance with the preserved neuronal shape and function in aged *C. elegans*, 50 µM BAM15 extended the mean lifespan of both wild-type and *ucp-4* mutants.

## 1. Introduction

Aging and age-related diseases have been highlighted because of the increasing elderly population worldwide [1]. Age-related diseases include neurodegenerative diseases such as Parkinson’s disease, Alzheimer’s disease, and Huntington’s disease. These neurodegenerative diseases are associated with dysfunctional mitochondria in the neuronal cells [2,3,4,5]. Studies have shown decreased mitochondrial bioenergetic function with age [6,7,8]. Moreover, increased levels of reactive oxygen species (ROS) and depleted ATP indicate unhealthy mitochondria [9]. During the progression of aging, responses to oxidative stress gradually fail by losing control of increased ROS [10]. Uncontrollable amounts of ROS can be generated by defective mitochondria via electron transport leakage [11,12]. Excessive ROS can also attack mitochondrial membrane lipids, proteins, and mitochondrial DNA, resulting in mitochondria with irreversible damage [13,14]. Since maintaining a healthy mitochondrial population is critical for proper functioning and survival in neuronal cells [15,16], research has been conducted on how to sustain the functional mitochondrial population, partly through mitophagy. One way is to use small protonophoric uncouplers such as carbonyl cyanide *p*-trifluoromethoxyphenylhydrazone (FCCP) via mitophagy [17]. Studies have shown that mitochondrial membrane uncoupling decreases ROS production [18,19,20], which is beneficial for maintaining a healthy mitochondrial population during aging. Small protonophore uncouplers have been extensively studied to harness several diseases, including age-related diseases [21]. Unfortunately, their therapeutic window is very narrow and the side effects are lethal [22].

Our previous work showed that defects in mechanosensory neuronal cells were alleviated in both uncoupling agent DNP-treated and uncoupling gene, *ucp-4,* overexpressed in *Caenorhabditis elegans* (*C. elegans*) during aging [23]. However, DNP is a controversial chemical because of its non-specific membrane depolarization and very narrow therapeutic window [24]. Recently, (2-fluorophenyl){6-[(2-fluorophenyl)amino](1,2,5-oxadiazolo [3,4-e]pyrazin-5-yl)}amine (BAM15) was identified as a protonophore uncoupler with fewer harmful characteristics [25]. BAM15 depolarizes the mitochondrial membrane but not the plasma membrane, which can minimize off-target side effects in vivo applications [25]. The characteristics of BAM15 could make it a great therapeutic agent for treating age-related diseases, including neurodegenerative diseases. Therefore, it is worth investigating BAM15 effects on both neurodegenerations during aging and on lifespan in vivo. *C. elegans* has been a great model animal for studying aging and age-associated neurodegenerative diseases owing to its short life span. In addition, its mechanical neurons can be easily manipulated and observed under a microscope. To date, no studies have investigated the effects of BAM15 on neuronal cells in vivo during aging. We investigated the relationship between BAM15-induced mitochondrial uncoupling and neurodegeneration during aging and between uncoupling and lifespan in *C. elegans*. Herein, we show that BAM15 reduces the abnormal shapes of mechanosensory neuronal cells and maintains the function of touch and short-term memory during aging, potentially via mitochondrial uncoupling, in *C. elegans*. Consistent with the beneficial effects on neurodegeneration, the lifespan of 50 µM BAM15-treated animals was longer than that of untreated controls. Taken together, BAM15 contributes to the health span and mean lifespan of *C. elegans*.

## 2. Materials and Methods

### 2.1. Strains and Culture

The Bristol N2 wild-type, *ucp-4* deletion mutant (*ok195*, CY121), and *zdIs5* ([*Pmec-4::GFP*], *CZ10175*) strains were provided by the Caenorhabditis Genetics Center (CGC) at the University of Minnesota. The *ucp-4* mutant was crossed with *zdIs5* to obtain *ucp-4*;*zdIs5* for observation of mechanosensory neurons. All strains were maintained in accordance with standard protocols [26].

In all experiments, a liquid culture system based on the Solis and Petrascheck protocol was used with slight modifications [27]. In brief, nematode eggs were synchronized and hatched on NGM-*E. coli* OP50 seeded plates. L1 and L2 stage larvae were collected, washed, and transferred to fresh NGM-*E. coli* OP50 plates until the L4 stage was reached. L4 nematode worms were collected and transferred to S-complete media with *E. coli* OP50 (4 × 10^9^ cells/mL). *E. coli* OP50 as a food source was prepared according to the paper by Solis and Petrascheck, 2011. *E. coli* OP50 was inoculated in 50 mL of TB buffer and incubated overnight at 37 °C. The following day, the overnight culture solution was poured into 500 mL of TB buffer and incubated for 3 h at 37 °C. OP50 bacteria were collected by centrifugation at 2200× *g*, 10 min. The bacterial pellets were washed with sterilized water and centrifuged again. The final bacteria pellets were dissolved by S-complete solution at the final concentration of 0.1 g/mL (approximately 2 × 10^10^ cells/mL).

To achieve synchronous populations of *C. elegans* and prevent internal egg hatching, fluorodeoxyuridine (FUDR) was applied to the liquid culture medium at a final concentration of 120 μM.

A 10 mM stock solution of 2,4-dinitrophenol (DNP; Sigma-Aldrich, Saint Louis, MO, USA) and a 10 mM stock of (2-fluorophenyl){6-[(2-fluorophenyl)amino](1,2,5-oxadiazolo[3,4-e]pyrazine-5-yl)}amine (BAM15; Sigma-Aldrich) were prepared in dimethyl sulfoxide (DMSO; Sigma-Aldrich). The stock solution was added to the S-complete medium at final concentrations of 10 µM and 50 µM.

### 2.2. Observation of Neuronal Defects

Morphological abnormalities of the anterior lateral microtubule (ALM) and posterior lateral microtubule (PLM) were counted under a fluorescence microscope (80i-DS-Fi1, Nikon). Outgrowth, waviness, blebbing, neuronal sprouts, and branching were scored as abnormal neurons [28,29,30,31]. Unpaired Student’s *t*-tests were performed to compare DNP- or BAM15-treated worms with DMSO controls. *p*-values < 0.05 were considered statistically significant.

### 2.3. Mechanosensory Neuron Assay-Touch Response

The touch response assay was performed according to a previous study [32]. In brief, nematode worms were cultured in a 25 mL culture bottle containing S-complete *E. coli* OP50 media. For the touch assay, 100 µL of culture solution (approximately 30 worms) was transferred to NGM solid media with seeded *E. coli* OP50. After 5 min, the individual animals were touched gently at the anterior half of the body using an eyebrow hair under a microscope. When animals backed away from the touch, it was scored as a positive response. The touches were repeated ten times, and the positive responses of the ten touches were presented as a ratio. Unpaired Student’s *t*-tests were performed to compare DNP- or BAM15-treated worms with DMSO controls. *p*-values < 0.05 were considered statistically significant.

### 2.4. Short-Term Associative Memory Assay

The nematodes were cultured in 25 mL cell culture bottles with S-complete *E. coli* OP50 media. The short-term memory assay was conducted as described by Margie et al. [33]. Approximately 1500 worms from each culture bottle were collected into 15 mL conical bottom tubes and washed three to four times in M9 buffer. Once the worms had precipitated, approximately 100 worms were transferred to an assay plate for naive chemotaxis (C_naive_). The remaining worms were then starved at room temperature for one hour. After starvation, the worms were conditioned in plates with OP50 seeded and 10% butanone. After 1 h of incubation at RT, a chemotaxis assay (C_0_, *t* = 0) was performed. The remaining worms were transferred onto fresh NGM-seeded *E. coli* OP50 plates. After an hour of incubation, the worms were washed and assayed for chemotaxis (C_1_, *t* = 1). The assay plates were marked and spotted as origin, butanone, and ethanol spots, according to the protocols [34,35,36]. The chemotaxis index (CI) was calculated as:

Chemotaxis index (CI) = [(number of worms at butanone) − (number of worms at ethanol)]/[(total number of worms on the plate) − (number of worms at origin)].

Learning Index (LI) = chemotaxis index at time-chemotaxis index at naive.

### 2.5. Lifespan Measurement

After 22 h of incubation from the L4 stage in S-complete media, the young adult worms were distributed into 96-well microtiter plates at a density of three to five worms per well. DNP and BAM15 were added to the liquid media at final concentrations of 10 µM and 50 µM, respectively, on day 1. Live and dead worms in each well were counted under a microscope every third day until all worms were dead.

### 2.6. Statistical Analysis

Data are reported as mean ± standard error of the mean (SEM) except lifespan analysis. Prism 9 software (GraphPad, San Diego, CA, USA) was used for statistical analysis. Lifespan analysis was performed using OASIS 2 [37].

## 3. Results

### 3.1. BAM15 Alleviates Neuronal Defects in Aged Wild-Type N2

**Figure 1:**
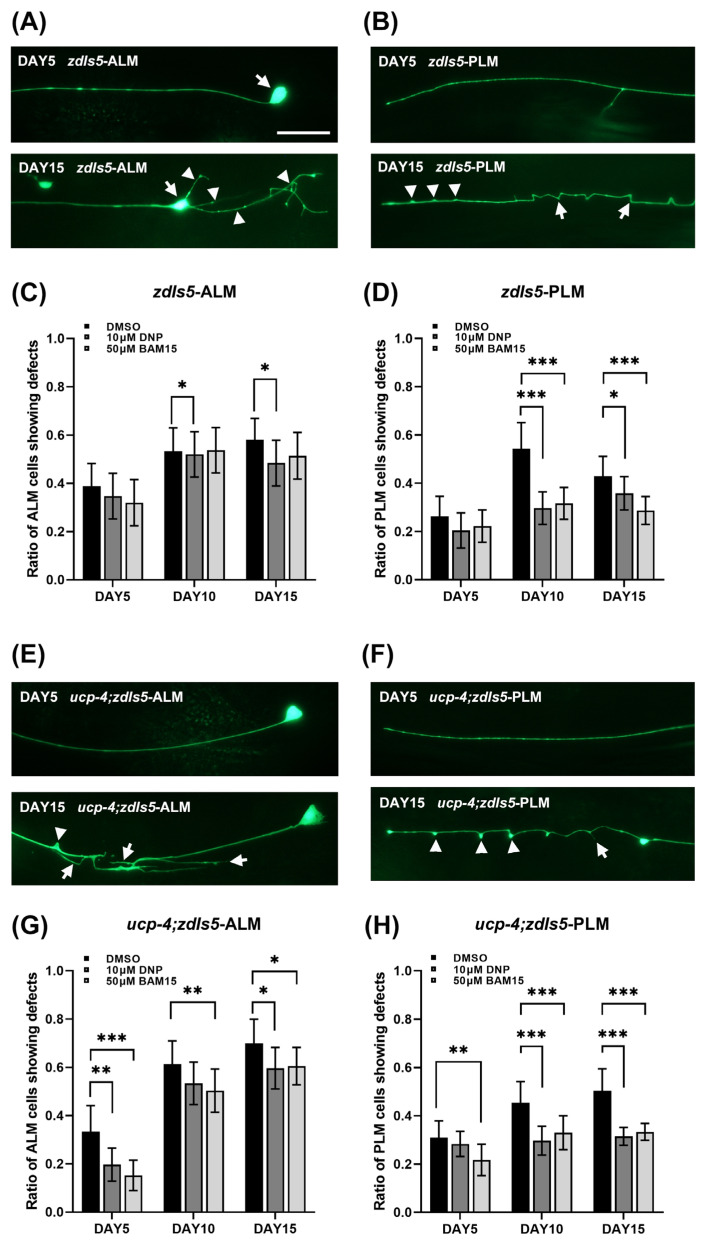
BAM15 attenuates neuronal defects in aged *C. elegans*. (**A**,**B**) Representative images of mechanosensory neurons in *zdIs5* [*Pmec-4::GFP*] animals on Day 1 and Day 15; ALM and PLM, respectively. Arrows indicate soma and arrowheads indicate soma outgrowth and branching in ALM (**A**). Arrows indicate blebs and arrowheads indicate wavy processing in PLM (**B**). Scale bar = 50 µm. (**C**,**D**) ALM and PLM neuronal defects are presented as a ratio of the total ALM- and PLM-scored neurons in animals treated with 10 µM DNP and 50 µM BAM15 on Days 5, 10, and 15. (**E**,**F**) Representative images of mechanosensory neurons in *ucp-4*;*zdIs5* animals on Days 1 and 15; ALM and PLM, respectively. Arrows indicate neuronal sprouting and arrowhead indicates a bleb in ALM (**E**). An arrow indicates branching and arrowheads indicate blebs (**F**). (**G**,**H**) ALM and PLM neuronal defects are presented as a ratio of the total ALM- and PLM-scored neurons in *ucp-4*;*zdIs5* animals treated with 10 µM DNP and 50 µM BAM15 on Days 5, 10, and 15. ALM, anterior lateral microtubules; PLM, posterior later microtubules. Data are represented as means of three independent experiments. Error bars represent the SEM. *n* = 80~120 worms per treatment in each experiment. * *p* < 0.05, ** *p* < 0.01, *** *p* < 0.001. Unpaired Student’s *t*-tests were performed to compare the DMSO control and treatments.

Previous studies have shown that aging deteriorates mechanosensory neurons, such as anterior lateral microtubule (ALM) and posterior lateral microtubule (PLM) cells. Mitochondrial uncoupling by DNP treatment or *ucp-4* overexpression decreases ALM and PLM neuronal defects in aged *C. elegans* [23]. Recently, Kenwood et al. identified and validated that (2-fluorophenyl){6-[(2-fluorophenyl)amino](1,2,5-oxadiazolo[3,4-e]pyrazin-5-yl)}amine (BAM15) was a mitochondrial uncoupler (Supplementary Figure S1). It has been suggested that BAM15 has no effect on plasma membrane dissociation. Therefore, we applied BAM15 to *C. elegans* during aging and scored the ALM and PLM defects to test whether mitochondrial uncouplers mitigate neurodegeneration during aging. GFP-labeled ALM and PLM were scored in the *zdIs5* [*Pmec-4::GFP*] transgenic strain. Figure 1 shows representative images of normal and abnormal neurons in the wild-type and *ucp-4* mutant (Figure 1A,B,E,F, respectively). Arrows and arrowheads indicate abnormal phenotypes of neuronal cells, such as soma outgrowth, neuronal sprouting, branching, bleb, and wavy processes (Figure 1A,B,E,F).

According to the BAM15 study, a 10 µM concentration showed potent uncoupling activity in the 0 to 50 µM application range [25]. Based on this study, we applied 10 µM and 50 µM BAM15 to wild-type animals during aging. The neuronal defects were more reduced in 50 µM BAM15-treated animals compared to 10 µM-treated animals (Supplementary Figure S2). Thereafter, the 50 µM concentration was used in all subsequent experiments. We scored neuronal defects in wild-type animals treated with 50 µM BAM15 on days 5, 10, and 15 after the adult stage (Figure 1C,D). An equal volume of DMSO to 50 µM BAM15 was applied to the animals as the negative control, and 10 µM DNP treatment was used as the positive control. At day 5, no differences in neuronal defects among all treatments were observed (Figure 1C,D). At day 10, neuronal defects in 10 µM DNP-treated animals were decreased compared to the DMSO control (16% reduction in ALM and 45% reduction in PLM). Animals treated with 50 µM BAM15 showed a significant reduction in PLM defects compared to the control, with a 41% reduction. However, no significant reduction was observed in the ALM defects. At day 15, animals treated with 10 µM DNP showed 16% reduced defects in both ALM and PLM, and PLM defects in 50 µM BAM15-treated animals were reduced by 32%. After 15 days, the scoring of neuronal defects was unreliable because almost all neurons contained at least one defect. The result in the wild-type indicates that aging-associated neurodegeneration is alleviated by the mitochondrial-specific uncoupler BAM 15.

### 3.2. BAM15 Attenuates Neuronal Defects in the Aged Ucp-4 Mutant

A previous study validated that neuronal defects are severe in uncoupling defect *ucp-4* mutants during aging, and the defects are reduced in uncoupler DNP- and CCCP-treated mutants [23]. Since BAM15 is specifically a mitochondrial membrane uncoupler, we investigated neuronal defects in aged *ucp-4* mutants with 50 µM BAM15 application. On day 5, a reduction in neuronal defects was observed in the *ucp-4* mutant treated with BAM15, a 54% reduction in ALM, and a 30% reduction in PLM (Figure 1G,H). During the aging progress, the BAM15-treated mutants demonstrated continuously reducing neuronal defects. ALM defects decreased by 18% and 14% on days 10 and 15, respectively, and PLM defects decreased by 27% on day 10 and 33% on day 15 compared to the defects in DMSO control mutants (Figure 1G,H). Consistent with a previous study, 10 µM DNP reduced ALM and PLM defects in *ucp-4* mutants during aging by 40% on day 5-ALM, 15% on day 15-ALM, 34% in day 10-PLM and 37% in day 15-PLM compared to the neuronal defects in the DMSO control. The results for the uncoupling mutant *ucp-4* suggests that BAM15 alleviates neurodegeneration in aged *C. elegans*, likely via mitochondrial uncoupling.

### 3.3. BAM15 Alleviates Loss of Touch Senses in Aged C. elegans

**Figure 2:**
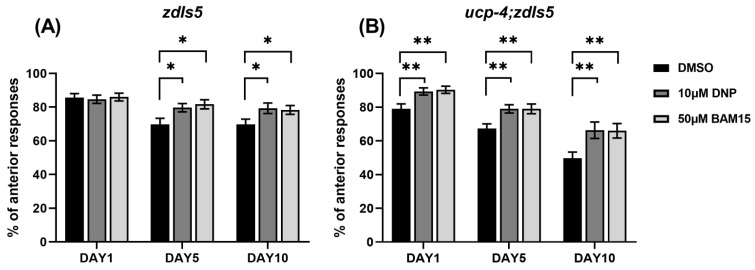
BAM15 retains touch responses in aged *C. elegans*. (**A**,**B**) Anterior touch responses are presented as a percentage of the total responses in *zdIs5* and *ucp-4*;*zdIs5* worms treated with 10 µM DNP and 50 µM BAM15 on Days 1, 5, and 10. Data are represented as means of three independent experiments. Error bars represent the SEM. *n* = 30 worms per treatment in each experiment. * *p* < 0.05, ** *p* < 0.01. Unpaired Student’s *t*-tests were performed to compare the DMSO control and treatments.

Jiang et al. showed a positive relationship between ALM morphology destruction and decreased touch responses in aged *C. elegans* [38]. To test the correlation between the abnormal shapes of mechanosensory neurons and the deterioration of neuronal function, we performed a gentle touch assay of ALM on the same days as the phenotypic neuronal scoring: days 5 and 10. We only scored ALM responses because PLM desensitized quickly after two to three stimulations. In addition, on day 15, even the anterior touch responses were very limited and irregular, regardless of treatment and genotype. Thereafter, we performed the touch assay for up to 10 days. There were no statistically significant differences in the anterior touch responses in all treatments of wild-type animals at day 1 (Figure 2A). As aging progressed, 10 µM DNP- and 50 µM BAM15-treated wild-type animals responded more than the DMSO control animals (14% and 17% at day 5 and 14% and 12% at day 10, respectively; Figure 2A).

In contrast to the wild-type, *ucp-4* mutants treated with 10 µM DNP and 50 µM BAM15 showed enhanced touch responses even at day 1 compared to the DMSO controls (13 and 14%, respectively; Figure 2B). At day 5 and day 10, progressive increments in touch responses were observed in both DNP and BAM15 treated *ucp-4* mutants (both 17% at day 5 and 33% at day 10, Figure 2B). These results suggest a positive relationship between phenotypic neuronal defects and functional deterioration in mechanosensory neurons in aged *C. elegans*.

### 3.4. BAM15 Enhances Short-Term Memory in Aged C. elegans

**Figure 3:**
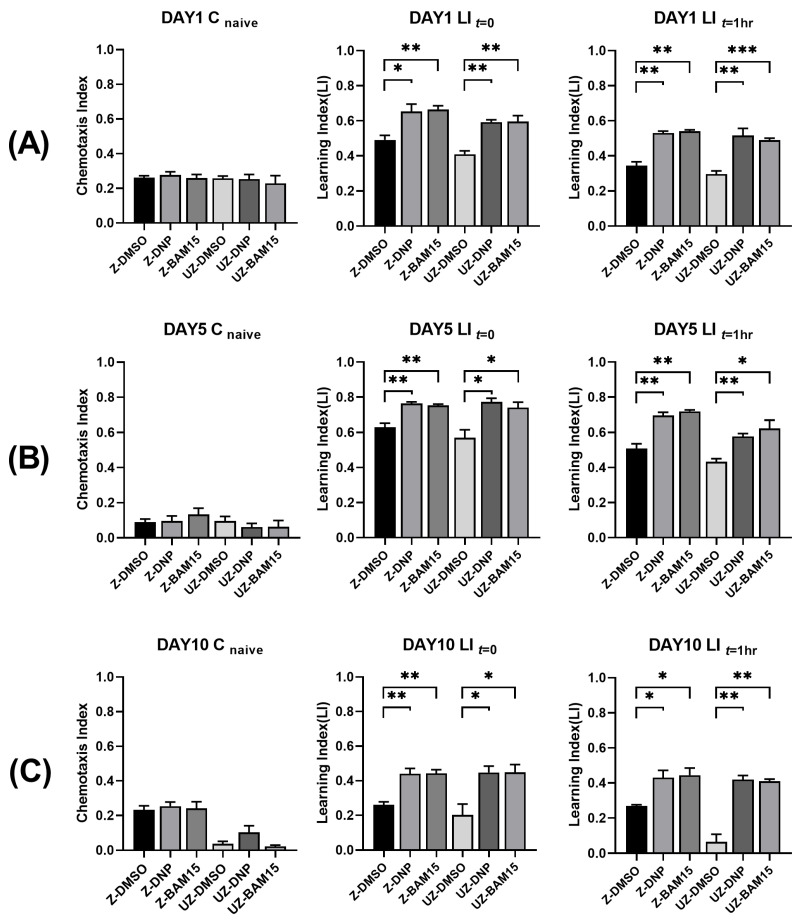
BAM15 retains learning ability and short-term memory in aged *C. elegans*. (**A**–**C**) Naive Chemotaxis Index (CI) and Learning Index (LI) in *zdIs5* and *ucp-4*;*zdIs5* treated with 10 µM DNP and 50 µM BAM15 on Day 1 (**A**), Day 5 (**B**), and Day 10 (**C**). *t* = 0, right after the training; *t* = 1 h, 1 h after the training. Z-DMSO—DMSO control in *zdIs5*; Z-DNP—10 µM DNP treatment in *zdIs5*; Z-BAM15—50 µM BAM15 treatment in *zdIs5*; UZ-DMSO—*ucp-4*;*zdIs5* treated with DMSO; UZ-DNP—*ucp-4*;*zdIs5* treated with 10 µM DNP; UZ-BAM15—*ucp-4*;*zdIs5* treated with 50 µM BAM15. Data are represented as means of three independent experiments. Error bars represent the SEM. *n* = 150~300 worms per treatment in each experiment. * *p* < 0.05, ** *p* < 0.01, *** *p* < 0.001. Unpaired Student’s *t*-tests were performed to compare the DMSO control and treatments.

Based on the above results, we investigated whether BAM15 reduced the cognitive functional decline in neurons. We performed a short-term associative memory assay using chemotaxis responses [35]. According to the protocol, we assayed the chemotaxis responses at three time points: naive (C_naive_), immediately after the training session (C_0_), and an hour after the training session (C_1h_). After one day of 10 µM DNP and 50 µM BAM15 application, chemotaxis responses toward butanone were enhanced in both DNP- and BAM15-treated wild-type and *ucp-4* mutant animals (Figure 3A). In the assay immediately after the training session, DNP- and BAM15-treated wild-type animals showed increased learning ability compared to non-treated control animals (33% and 35%, respectively). The *ucp-4* mutant also showed similar results, with a 45% enhanced learning ability in both DNP- and BAM15-treated ones. In the hour after the assay after training, DNP- and BAM15-treated animals retained their learning abilities. The learning index in the DNP- and BAM15-treated wild-type was 50% higher than that in the control wild-type (Figure 3A). In the *ucp-4* mutant, learning ability in 10 µM DNP-treated and 50 µM BAM15-treated animals was 74% and 65%, respectively, which is higher than that in DMSO-treated mutants (Figure 3A).

To determine whether DNP and BAM15 assisted in maintaining memory during aging, we assayed chemotaxis on days 5 and 10. On both day 5 and day 10, enhanced learning and memory in DNP- and BAM15-treated animals were observed (Figure 3B,C). At day 5, both DNP and BAM15 enhanced learning ability by 20% compared to the DMSO control in the wild-type, and *ucp-4* mutants treated with DNP and BAM15 showed 36% and 30% increased learning ability, respectively, compared to the DMSO control (Figure 3B). One hour after training, wild-type animals treated with both DNP and BAM15 showed increased learning ability compared with the DMSO control (both 41%, Figure 3B). In the *ucp-4* mutant treated with DNP and BAM15, a 33% and 44% increase in learning ability was observed, respectively, compared to the DMSO control (Figure 3B). At day 10, the learning ability of the DNP- and BAM15-treated wild-type was much greater than that of the DMSO control, and the *ucp-4* mutant treated with both uncouplers also showed greater increased ability compared to the control mutant (Figure 3C). Even after an hour, the wild-type treated with uncouplers showed almost two-fold enhanced responses relative to the control. Moreover, the *ucp-4* mutant treated with uncouplers showed more than six times higher ability than the control animals (Figure 3C). The increased learning index on day 5 and day 10 suggests that the uncouplers DNP and BAM15 assist in maintaining learning ability and memory in aged *C. elegans*.

### 3.5. 50 µM BAM15 Extends Mean Life Span in the Wild-Type and Ucp-4 Mutant

**Figure 4:**
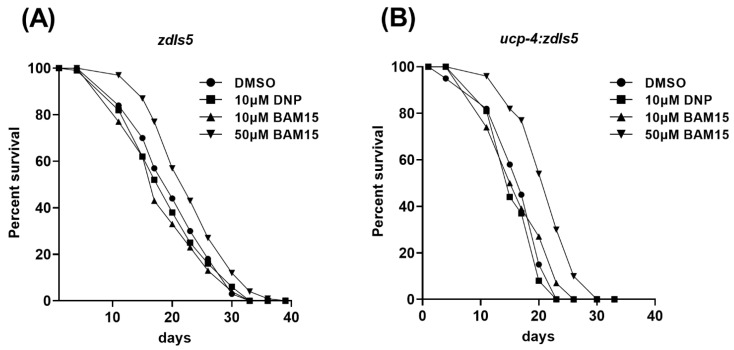
BAM15 extends the mean lifespan in *C. elegans*. (**A**,**B**) Kaplan–Meier survival curves of *zdIs5* (**A**) and *ucp-4*;*zdIs5* (**B**) treated with 10 µM DNP, 10 µM BAM15, and 50 µM BAM15. *n* = 248~309 per treatment in each genotype. Log-rank test, *p* < 0.001 between DMSO and 10 µM DNP, and DMSO and 50 µM BAM15 treatments. Data are presented as means of three independent experiments.

Mitochondrial uncoupling is thought to extend the lifespan [39]. Hence, we tested whether BAM15 increased the mean lifespan of *C. elegans*. Because lifespan can be an accumulative result of long periods of exposure to the chemical, we also included a 10 µM BAM15 treatment. Unexpectedly, the mean lifespan in 10 µM DNP- and 10 µM BAM15-treated wild-type animals was slightly decreased (4% and 8%, respectively; Figure 4A and Table 1). In contrast, 50 µM BAM15-treated animals showed a 14% increase in mean lifespan (Figure 4A and Table 1). The *ucp-4* mutant also behaved as the wild-type, with a slightly reduced lifespan in 10 µM DNP- and 10 µM BAM15-treated mutants (Figure 4B and Table 1). Consistent with the wild-type lifespan expansion, the mean lifespan in the 50 µM BAM15-treated mutant was significantly extended by 28% (Figure 4B and Table 1). These results suggest that 50 µM BAM15 extends the mean lifespan of *C. elegans* with implications for mitochondrial uncoupling involvement.

## 4. Discussion

Many parameters are used as indicators of aging, and phenotypical and/or functional defective neurons are signs of aging [40,41,42]. Mechanosensory neurons in *C. elegans* are a good phenotypic indicator of neuronal deterioration because they are easy to observe under a microscope. To compare the functional aspects of neurodegeneration, we measured touch responses. The results of the touch assay indicated a positive correlation between phenotypic and functional deterioration during aging. One of the most devastating symptoms of deterioration in human aging is neurodegenerative decline, expressed as a loss of cognitive function [40]. Therefore, in addition to the mechanosensory touch assay, we measured the cognitive function of the neurons. The *C. elegans* chemotaxis assay, which is based on the nematode’s attraction to butanone, was used for this measurement [35]. As aging progresses, short-term memory decreases, indicating decreased neuronal cognitive function. Furthermore, the uncoupling mutant *ucp-4* showed greater memory reduction during aging, and uncoupler BAM15-treatments recovered the memory of *ucp-4* as the ability of the wild-type (Figure 3). These data suggest that mitochondrial uncoupling has a beneficial effect on maintaining neuronal function during aging. This is consistent with other studies showing that uncoupler DNP protects neuronal function [43,44].

We observed fewer neuronal defects in the PLM than in the ALM in all animals during aging, regardless of treatment (Figure 1C,D,G,H). However, the number of PLM defects was higher than that of ALM defects in our previous study [23]. The study was performed on NGM plates, and the current study was performed in liquid media, which may reduce mechanical stress [45]. Crawling on solid media requires more bending amplitude than swimming in liquid [46]. Nematode bending induces the activity of motor neurons and muscles in the posterior part of the bending [47]. It has been suggested that the backward movement of the nematode may generate more force and power than the forward movement [48]. Backward movement often correlates with escape from the predator, which requires maximum force and speed. Therefore, fewer PLM defects could result from mild-force swimming movements in liquid culture conditions.

Uncoupling with BAM15 enhances mitophagy and mitochondrial quality control in mouse skeletal muscle [49]. We also tested whether BAM15 is involved in mitophagy using a mitophagy-defective *pink-1/Parkin* double mutant. Unfortunately, the results showed no indication of different behaviors between wild-type and *pink-1/Parkin* double mutants (data not shown). In addition, mitophagy tracking using mitophagy markers in vivo is not an easy task despite many useful fluorescent-tagging tools [50]. The development of a tool for tracking mitophagy in *C. elegans* in vivo may be necessary prior to any further experiments. In a future study, BAM15 involvement in mitophagy in *C. elegans* could be tested using the mitophagy-related gene and/or protein expression profile.

Our results showed that 50 µM concentration of BAM15 is effective for neurodegeneration and lifespan, which is slightly higher than the concentration used in the obesity study [51]. According to the study, up to 40 µM BAM15 did not induce caspase 3/7 activation, an indicator of apoptosis. The 50 µM effective concentration in our study could be attributed to the whole-organism application rather than a single cell. In the same study, 5 µM DNP and 10 µM FCCP induced Caspase 3/7 activation in mouse myoblast cells [51]. However, we obtained the same result that 10 µM DNP- and 50 µM BAM15-treated animals showed no phenotypic and functional differences in neuronal defects. Consequently, we concluded that 10 µM DNP effectively reduced neurodegeneration without causing severe side effects in aged *C. elegans*. The potency of 10 µM DNP without severe side effects is consistent with our previous study, which accords with the recent development of DNP as a feasible neurodegenerative medicine [52].

In summary, we demonstrated that neurodegeneration in aged *C. elegans* was mitigated by the mitochondrial uncoupler BAM15 using the wild-type and *ucp-4* mutant. The mitochondrial uncoupler maintained the function of mechanosensory neurons and memory in the wild-type and *ucp-4* mutant during aging. Accordingly, BAM15 extended the mean lifespan of the wild-type and *ucp-4* mutant. BAM15 is an efficacious mitochondrial uncoupler without severe side effects and reduces neurodegeneration in aged *C. elegans* potentially via mitochondrial uncoupling. Further study of BAM15 mitochondrial uncoupling activity in *C. elegans* is necessary. Considering the neuronal aspect, BAM15 can enhance the health span and lifespan. Generally, it makes BAM15 a prime candidate for a medicine to treat aging-related diseases, including neurodegenerative diseases.

## Figures and Tables

**Table 1:** Summary of mean lifespan in both *zdIs5* and *ucp-4*;*zdIs5* treated with 10 µM DNP, 10 µM BAM15, and 50 µM BAM15. The values were derived from the survival curves. *n* = 248~309 per treatment in each genotype. Log-rank test, *p* < 0.001 between DMSO and 10 µM DNP, and DMSO and 50 µM BAM15 treatments. Data are presented as means of three independent experiments. NS, not statistically significant.

Genotype Treatments	Mean Lifespan (Days)	% Change in Mean Lifespan	*p*-Value
*zdIs5* DMSO	20.59 ± 0.30		
10 μM DNP	19.75 ± 0.48	−4	NS
10 μM BAM15	18.94 ± 0.58	−8	0.0335
50 μM BAM15	23.48 ± 0.59	+14	0.0003
*ucp-4*;*zdIs5* DMSO	17.00 ± 0.42		
10 μM DNP	16.51 ± 0.43	−3	NS
10 μM BAM15	17.12 ± 0.51	0	NS
50 μM BAM15	21.76 ± 0.46	+28	0.0

## Data Availability

The data presented in this study are available in this article and the Supplementary Materials.

## Supplementary Materials

The following supporting information can be downloaded at: https://www.mdpi.com/article/10.3390/metabo12111129/s1. Figure S1: Chemical structure and properties of BAM15 as a mitochondrial uncoupler. The acid dissociation constant (pKa) and LogP value are presented. DNP, 2,4-dinitrophenol; BAM15, (2-fluorophenyl){6-[(2-fluorophenyl)amino](1,2,5-oxadiazolo[3,4-e]pyrazine-5-yl)}amine. Figure S2: 50 µM BAM15 reduced more neuronal defects than 10 µM BAM15.
