# Supplementary material for: BAM15 Relieves Neurodegeneration in Aged Caenorhabditis elegans and Extends Lifespan
Source: Metabolites. 2022 Nov 17;12(11):1129. doi: 10.3390/metabo12111129 (PMC9698188; doi:10.3390/metabo12111129)
Supplement: Supplementary file 1 [file metabolites-12-01129-s001.zip › metabolites-1972660-supplementary.pdf]

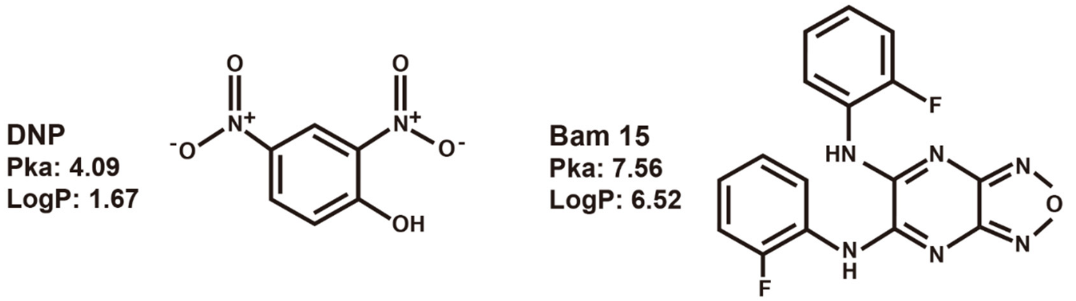

Supplement Figures 1: Chemical structure and properties of BAM15 as a mitochondrial uncoupler. The acid dissociation constant (pKa) and LogP value are presented. DNP, 2,4-dinitrophenol; BAM15, (2-fluorophenyl){6-[(2-fluorophenyl)amino](1,2,5-oxadiazolo[3,4-e]pyrazine-5-yl)}amine.

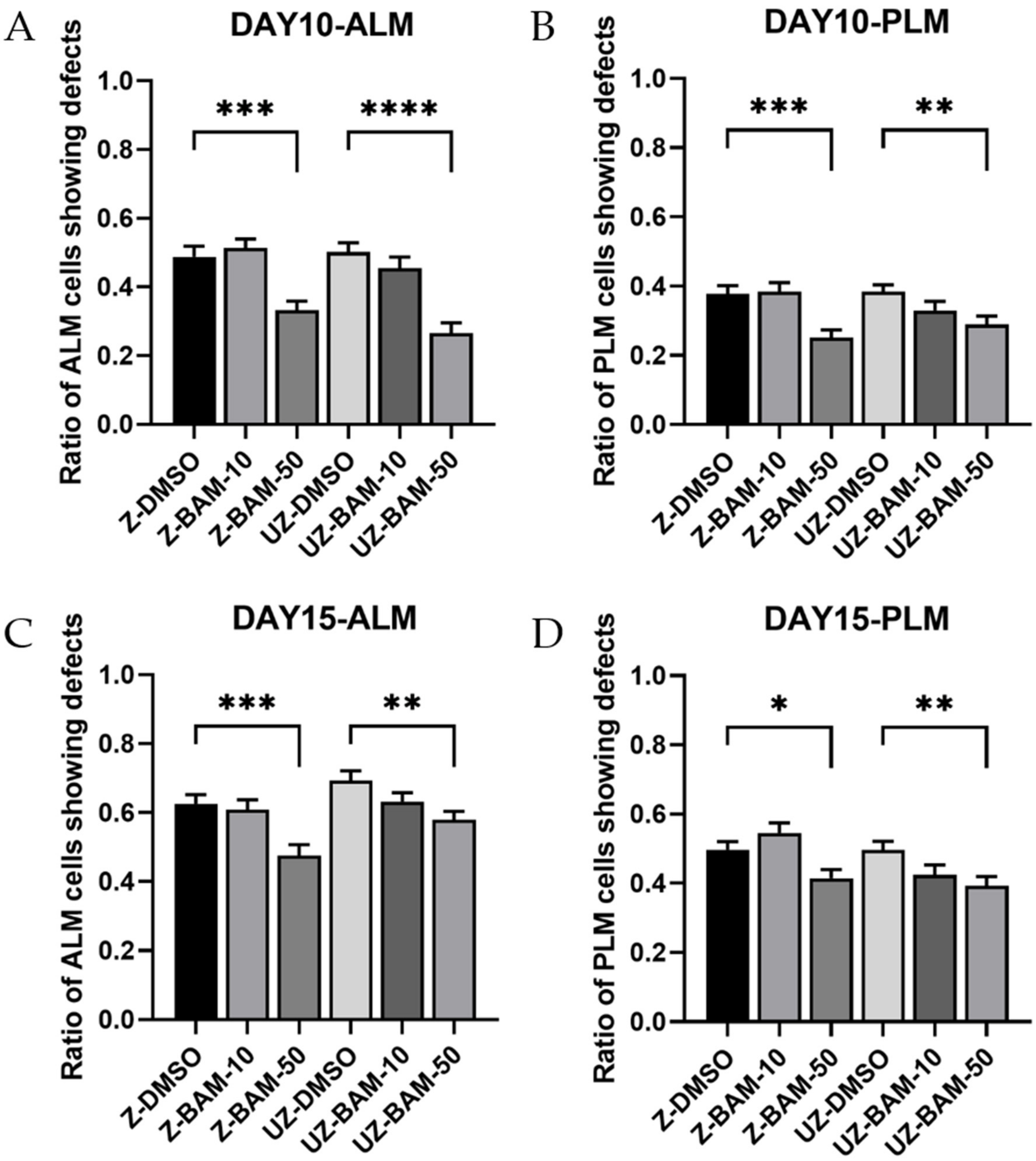

Supplement Figures 2. 50 μM BAM15 reduced more neuronal defects than 10 μM BAM15. (A–D) ALM and PLM neuronal defects are presented as a ratio of the total ALM- and PLM- scored neurons in animals treated with 10 μM BAM15 and 50 μM BAM15 on day-10 (A and B) and 15 (C and D). Z-DMSO, DMSO control in *zdIs5*; Z-BAM-10, 10 μM BAM15 treatment in *zdIs5*; Z-BAM-50, 50 μM BAM15 treatment in *zdIs5*; UZ-DMSO, *ucp-4*;*zdIs5* treated with DMSO; UZ-BAM-10, *ucp-4*;*zdIs5* treated with 10μM BAM15; UZ-BAM-50, *ucp-4*;*zdIs5* treated with 50μM BAM15. Error bars represent the SEM. n = 80~100 worms per treatment in each experiment. *$p<0.05$, **$p<0.01$, ***$p<0.001$, ****$p<0.0001$. Unpaired Student's t-tests were performed to compare DMSO control and treatments.
